# Supplementary material for: Pilot Study on the Impact of Polymorphisms Linked to Multi-Kinase Inhibitor Metabolism on Lenvatinib Side Effects in Patients with Advanced Thyroid Cancer
Source: Int J Mol Sci. 2023 Mar 13;24(6):5496. doi: 10.3390/ijms24065496 (PMC10049548; doi:10.3390/ijms24065496)
Supplement: Supplementary file 1 [file ijms-24-05496-s001.zip › ijms-2230005-supplementary.pdf]

Table S1. Primer sequences and PCR conditions

Genes	SNPs	Forward (5’-3’)	Reverse (5’-3’)	PCR conditions
CYP3A4	rs2687116	CTCCACTCAGCGTCTTTGGG	TGGTTGCATATGATGACAGGGT	60°C fast, 1.5 mM $MgCl_2$
	rs2242480	CTTAGGGATTTGAGGGCTTCA	TTCTCCTGGGAAGTGGTGAG	60°C fast, 1.5 mM $MgCl_2$
ABCB1	rs2032582	ATAGGTTCCAGGCTTGCTGT	ACTGGCTTTGCTACTTTCTGT	60°C fast, 1.5 mM $MgCl_2$
	rs1045642	GTGTGCTGGTCCTGAAGTTG	AAGGGTGTGATTTGGTTGCT	60°C fast, 1.5 mM $MgCl_2$
	rs2235048	GTGTGCTGGTCCTGAAGTTG	AAGGGTGTGATTTGGTTGCT	60°C fast, 1.5 mM $MgCl_2$
ABCG2	rs2231142	GGCTTTGCAGACATCTATGGA	CACATTACCTTGGAGTCTGCC	60°C fast, 1.5 mM $MgCl_2$
CYP3A5	rs776746	TGGAGAGTGGCATAGGAGAT	TGGATGCTTACCCTTCGATTTG	60°C fast, 1.5 mM $MgCl_2$
